# Using contrast-enhancing software to improve sentinel lymph node detection with indocyanine green in colon cancer surgery

**DOI:** 10.1007/s10151-026-03320-w

**Published:** 2026-05-13

**Authors:** Bart C. T. van de Laar, Daan J. Sikkenk, Bloem N. van Dam, Paul M. Verheijen, Wouter B. Nagengast, Esther C. J. Consten

**Affiliations:** 1 Department of Surgery, University of Groningen, University Medical Center Groningen, Hanzeplein 1, 9713 GZ Groningen, the Netherlands; 2 Department of Surgery, Meander Medical Center, Maatweg 3, 3813 TZ Amersfoort, the Netherlands; 3 Technical Medicine, University of Twente, Hallenweg 5, 7522 NH Enschede, the Netherlands; 4 Department of Gastroenterology and Hepatology, University of Groningen, University Medical Center Groningen, Hanzeplein 1, 9713 GZ Groningen, the Netherlands

**Keywords:** Fluorescence-guided surgery, Contrast-enhancing software, Indocyanine green, Sentinel lymph node biopsy, Robotic surgical procedures, Colonic neoplasms

## Abstract

**Background:**

Colon cancer (CC) is primarily treated with segmental resection, a surgical procedure associated with high morbidity and potential mortality. Sentinel lymph node (SLN) biopsy using the near-infrared fluorescence (NIRF) tracer indocyanine green (ICG) may help mitigate these risks by enabling organ-preserving surgery without the need for an anastomosis. It is hypothesized that contrast-enhancing software for NIRF could further improve SLN detection.

**Methods:**

This retrospective study evaluated whether a contrast-enhanced NIRF view improved SLN detection compared to the standard NIRF view of the robotic surgical system (da Vinci Xi). The contrast-enhanced view was generated using surgical image analysis software (PerfusionWorks), which enhances NIRF visual contrast. A retrospective analysis was conducted using intraoperative videos from SLN mapping with ICG in 10 patients with CC. Fifteen clinicians with varying levels of experience annotated video clips using both the standard and the contrast-enhanced views (44 clips per clinician in total). The sensitivity, specificity, accuracy, time-to-detection, and confidence scores were compared between the two views.

**Results:**

The contrast-enhanced view significantly improved sensitivity (49.3% to 62.4%, *p* < 0.001) and accuracy (46.1% to 51.8%, *p* < 0.001) compared to the standard view, with a decrease in specificity that was not statistically significant (39.4% to 30.4%, *p* = 0.08). On a 6-point Likert scale, clinicians reported higher confidence in their annotations with the contrast-enhanced view (median 5 vs. 4, *p* < 0.001).

**Conclusion:**

The contrast-enhancing software improved SLN detection in this test setting, improving sensitivity and accuracy. These findings support further investigation of its application in an intraoperative setting.

## Introduction

An increasing number of local excisions of T1 cancers are performed following the initiation of population screening for colorectal cancer [1]. However, local excisions are limited by the inability to assess lymph node status. Therefore, segmental resection including the resection of regional lymph nodes remains the main curative treatment for colon cancer (CC), despite the low incidence of lymph node metastasis in T1–2 CC (5–20%) [2–8]. However, segmental resections result in substantial morbidity and a perioperative mortality rate of 1.7–3.1% [9–12].

Alternatively, combining local treatment of the tumor with a sentinel lymph node (SLN) biopsy could potentially be a novel curative option for patients with early-stage CC [13, 14]. This organ-preserving surgery would provide nodal staging by selectively removing the first draining lymph nodes, rather than performing a complete lymphadenectomy. This approach eliminates the need for constructing an anastomosis, thereby reducing morbidity [15–20]. The near-infrared fluorescence (NIRF) tracer indocyanine green (ICG) has been proposed as a more effective tracer for SLN biopsy in CC compared to conventional dyes or radiocolloid, as ICG has demonstrated good tissue penetration and is more easily visualized in mesocolic fat [21, 22].

Currently, SLN detection and biopsy using ICG rely solely on the visual assessment of the NIRF view provided by the laparoscopic camera system. Ideally, the SLN would exhibit high-intensity fluorescence while the surrounding tissue remains non-fluorescent. However, this is not achievable with ICG and current camera systems, as the surrounding tissue also becomes fluorescent after ICG is injected into the peritumoral area and near the SLNs. Additionally, scattering, tissue autofluorescence, and the automatic gain settings of NIRF imaging systems further complicate this process [23]. Consequently, NIRF contrast-enhancing software may help surgeons detect and remove SLNs more effectively by improving contrast between SLNs and surrounding tissue. If visual enhancement improves SLN detection, it could be beneficial for intraoperative SLN biopsy.

Therefore, this pilot study aims to retrospectively assess whether contrast-enhanced software (PerfusionWorks, Perfusion Tech ApS, Copenhagen, Denmark) improves SLN detection in CC compared to the standard fluorescence visualization of the da Vinci Xi surgical robot (Intuitive Surgical Inc., Sunnyvale, USA), using previously acquired surgical videos of SLN mapping with ICG in patients with CC [24].

## Methods

### Video material

Intraoperative videos of 10 in vivo SLN mapping procedures were previously recorded. The procedures were carried out as described earlier [24]. In brief, the da Vinci Xi surgical platform was used for surgery. Ten patients with cT1–2N0M0 CC underwent intraoperative colonoscopy with submucosal injection of 1 ml of 5 mg/ml ICG in four aliquots around the tumor (4 ml in total). After injection of ICG, the mesocolon was inspected using the NIRF Firefly mode of the da Vinci Xi. SLNs (defined as the first, up to four, fluorescent lymph nodes visible after injection) were marked with a suture and standard-of-care segmental resection was subsequently performed. From these video recordings, 22 anonymized shorter video clips (range 9–35 s) were extracted which showed moments of ICG visualization with the NIRF camera. A total of 23 SLNs could be found in 16 video clips, while no SLNs were visible in 6 video clips. For each video clip, a standard NIRF view using the Firefly mode and a contrast-enhanced view was available, resulting in 44 video clips with 46 SLNs to be assessed.

### Study design

**Fig. 1:**
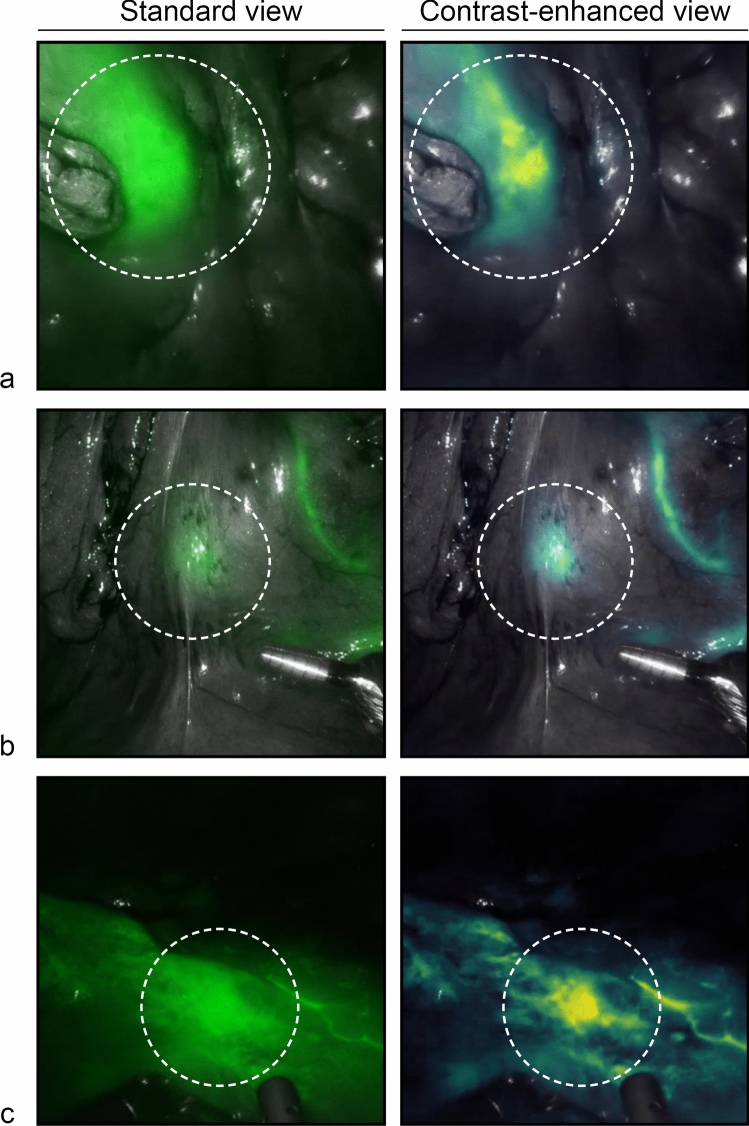
**a**–**c** Corresponding video frames of three different SLNs, shown in both the standard view and the contrast-enhanced view. SLNs are highlighted with white dotted circles

A cohort of 15 clinicians, including surgeons and surgical residents with varying levels of experience, consented to participate in this study. None had previously viewed the SLN mapping videos. Each clinician received written instructions outlining the study tasks. The video clips were presented in a randomized order, and each clinician assessed both the standard and contrast-enhanced views of every video clip, resulting in a fully paired design with two groups (Fig. 1). There were two distinct tasks: in the first task, clinicians analyzed 24 video clips, where the video automatically paused on the last frame for annotation. They then identified and annotated SLN locations, if present. In the second task, clinicians manually paused the video and annotated the visible SLNs as soon as they became detectable in the remaining 20 video clips. They were informed that some videos did not contain visible SLNs, in which case no annotations should be made. Each annotation was assigned a confidence score using a 6-point Likert scale, with 1 indicating very low confidence and 6 indicating very high confidence in their decision. Performance was compared between surgeons and residents, as well as between surgical oncologists and other clinicians. Before starting the assessments, all clinicians completed three test cases under supervision to ensure they understood the instructions: two with a visible SLN and one without.

### Outcomes

All fluorescent hotspots in the video clips were compared to pathological findings from the previously mentioned study, which served as the ground truth [24]. Clinicians’ annotations were evaluated for correctness by comparing them to the ground truth. Annotations were marked as true positive or false positive. If a video clip was not annotated, it was marked as true negative or false negative. Subsequently, sensitivity, specificity, and accuracy rates were calculated for both the standard and contrast-enhanced views. In the second task, where clinicians manually paused the video to annotate visible SLNs as quickly as possible, time-to-detection was recorded. Confidence scores were recorded for all true positive and false positive annotations.

### Contrast-enhancing software

PerfusionWorks, an image analysis software package developed for intraoperative quantitative ICG assessment, was used as the contrast-enhancing software [25]. PerfusionWorks can also be utilized to retrospectively analyze recorded videos. One of its features is a NIRF contrast-enhanced overlay, which, when activated during surgery, provides the clinician with a contrast-enhanced alternative view of the surgical video feed in real time on a secondary screen. The contrast-enhancement is achieved through a proprietary method developed by Perfusion Tech, for which a patent is pending.

To generate paired contrast-enhanced versions of the original surgical videos, the videos were opened in the software and saved with the contrast-enhanced overlay activated. Subsequently, a research version of the software was used by participating clinicians to annotate and score their confidence levels for both the standard and contrast-enhanced views of the original videos, as described earlier.

**Fig. 2:**
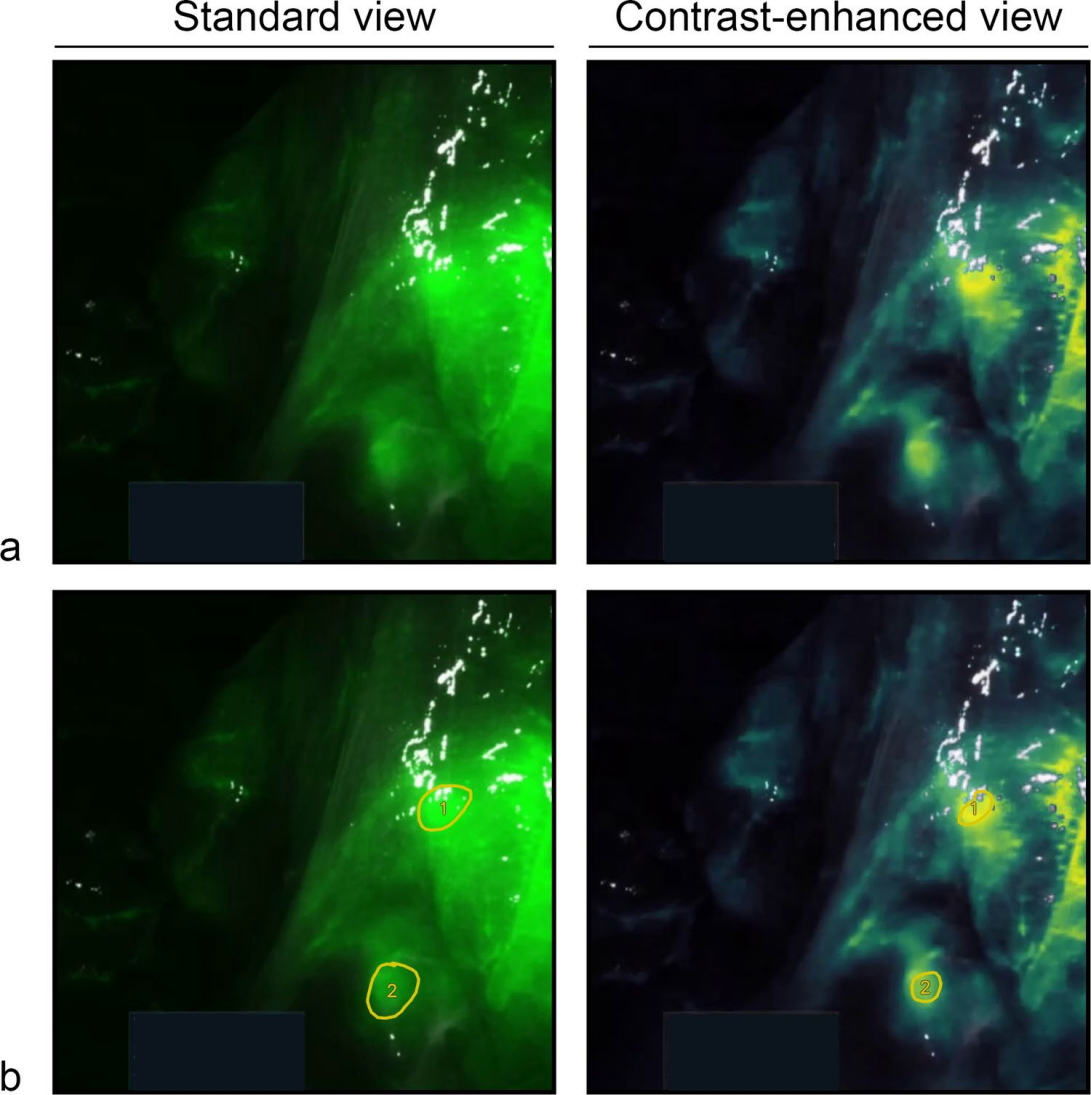
**a** Two SLNs against background noise in both the standard view and the contrast-enhanced view. **b** Examples of true positive annotations made on the touchscreen tablet (yellow circles)

All annotations were made on a touchscreen tablet (Microsoft Surface Pro 8) running Windows 11 Pro and with the research version of the software installed. Clinicians drew annotations by hand using a touchscreen stylus (Fig. 2b).

### Definitions

Sensitivity was determined as the ratio of true positive annotations to the sum of true positives and false negatives. Specificity was calculated as the ratio of true negatives to the sum of true negatives and false positives. Accuracy was defined as the proportion of all correct findings (true positives and true negatives) relative to all findings (true positives, true negatives, false positives, and false negatives). Time-to-detection was defined as the number of seconds until a true positive was annotated. For cases where no (correct) annotation was made despite a visible SLN (false negative), the duration of the full-length video clip in seconds was recorded as time-to-detection.

### Statistical analysis

Statistical analysis was performed using McNemar’s test to evaluate differences in sensitivity, specificity, and accuracy between the standard and contrast-enhanced video groups. The Mann–Whitney* U* test was used to assess differences in confidence scores and the paired* t* test was used to compare time-to-detection. A* p* value of < 0.05 was considered significant. Clinicians were divided into subgroups based on their experience as either surgeons or residents. Additionally, clinicians were categorized as either surgical oncologists or other clinicians, with the latter group consisting of other surgeons and residents. All analyses were performed using Python (version 3.9.13).

## Results

### Participating clinicians

All 15 clinicians, comprising 10 surgeons and five surgical residents, provided oral consent to participate. Among the surgeons, seven were surgical oncologists with experience in colorectal cancer surgery and/or SLN biopsies for breast cancer and melanoma. All clinicians completed the test cases and assessments without interruptions.

### Sensitivity, specificity, and accuracy rates

**Fig. 3:**
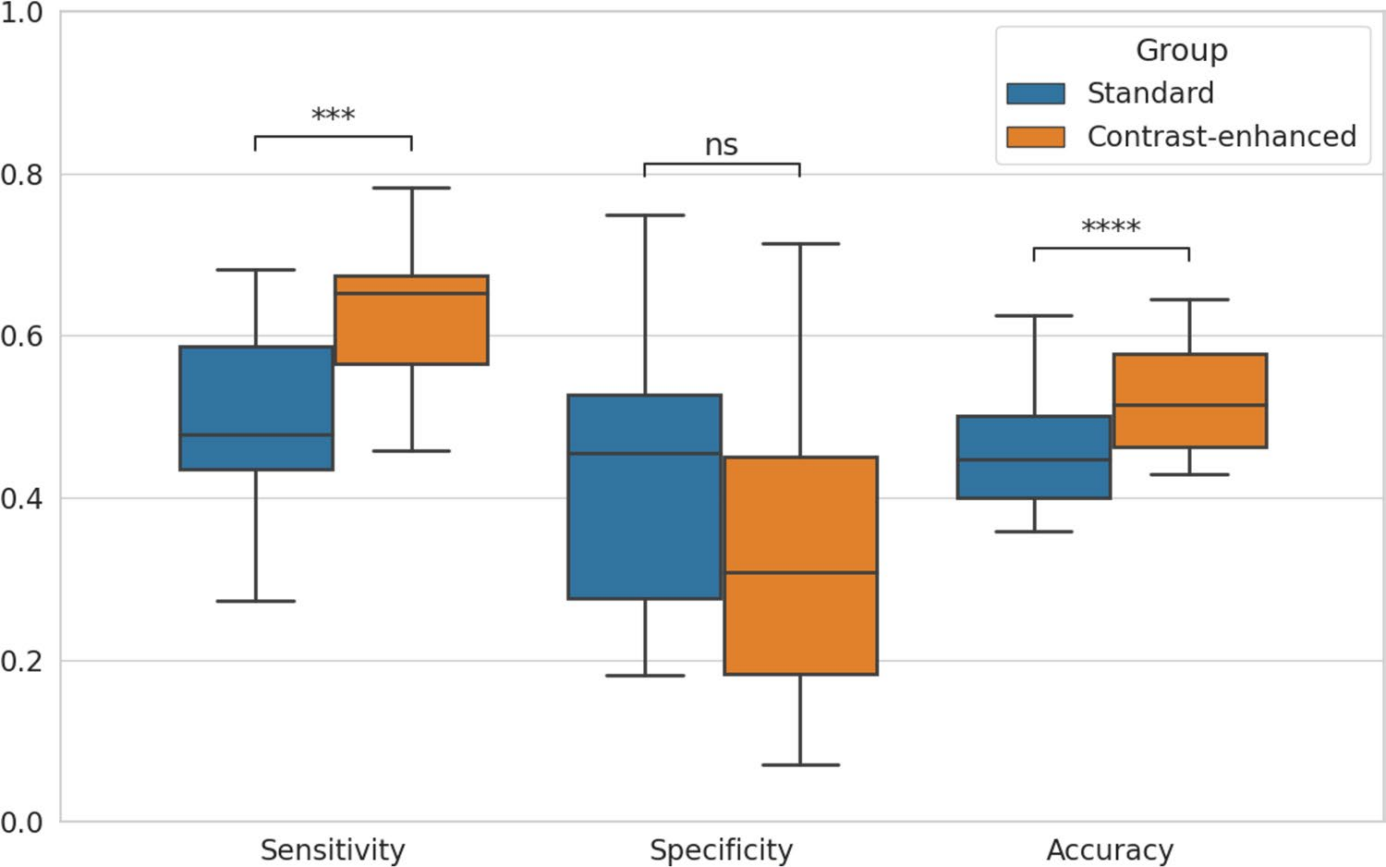
Boxplot of sensitivity, specificity, and accuracy rates for the standard versus contrast-enhanced views. McNemar’s test, ***p < 0.001, ****p < 0.0001,* ns* non-significant

In total, 1020 annotations were made, with a mean of 68 per clinician. Sensitivity improved from 49.3% (95% CI 44.0–54.5) with the standard view to 62.4% (95% CI 57.2–67.4) with the contrast-enhanced view (*p* < 0.001). Specificity decreased from 39.4% (95% CI 32.1–47.1) to 30.4% (95% CI 24.0–37.8) with the contrast-enhanced view, but this difference was not statistically significant (*p* = 0.08). Accuracy also increased from 46.1% (95% CI 41.8–50.5) to 51.8% (95% CI 47.5–56.1) with the contrast-enhanced view (*p* < 0.001). In all subgroups, namely surgeons, residents, surgical oncologists, and other clinicians, sensitivity and accuracy increased with the contrast-enhanced view (*p* < 0.05), while specificity decreased. However, the decrease in specificity was not statistically significant. Furthermore, clinicians reported a higher median confidence when annotating with the contrast-enhanced view compared to the standard view (5 vs. 4, *p* < 0.001). In the standard view, both true positives and false positives were annotated with a median confidence score of 4. In contrast, in the contrast-enhanced view, true positives were annotated with a median confidence score of 5, while false positives had a median confidence score of 4. The performance of detecting SLNs in the standard and contrast-enhanced views within the subgroups is summarized in Table 1 and Fig. 3.

**Table 1:** Sensitivity, specificity, accuracy rates, and median confidence scores for the standard and contrast-enhanced views

Group	Sensitivity		Specificity		Accuracy		Median confidence	
	Standard	Contrast-enhanced	Standard	Contrast-enhanced	Standard	Contrast-enhanced	Standard	Contrast-enhanced
All (*N* = 15)	0.493*	0.624*	0.394	0.304	0.461*	0.518*	4^#^	5^#^
Surgeons (*N* = 10)	0.518*	0.657*	0.398	0.283	0.480*	0.534*	4	5
Residents (*N* = 5)	0.447*	0.560*	0.386	0.345	0.427*	0.489*	4	5
Surgical oncologists (*N* = 7)	0.538*	0.683*	0.444	0.338	0.509*	0.574*	4	5
Other clinicians (*N* = 8)	0.454*	0.573*	0.352	0.278	0.421*	0.472*	4	5

*McNemar’s test with a difference between the standard and contrast-enhanced views with *p* < 0.05

^#^Mann–Whitney* U* test with a difference between the standard and contrast-enhanced views with *p* < 0.05

### Time-to-detection

The mean time-to-detection was 17.6 s for the standard view and 16.2 s for the contrast-enhanced view, with no statistically significant difference observed (*p* = 0.24).

### Surgical oncologists versus other clinicians

**Fig. 4:**
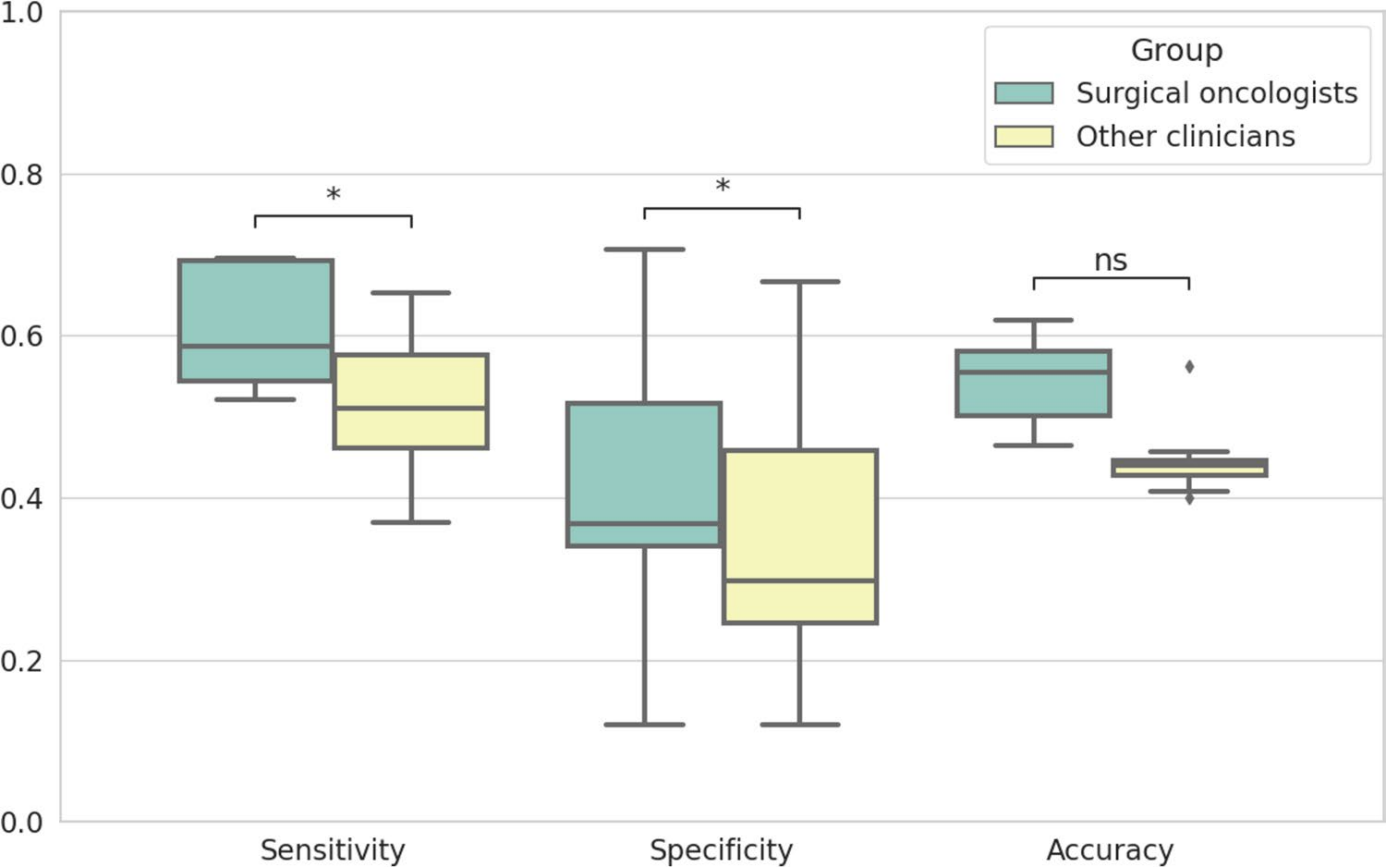
Boxplot of sensitivity, specificity, and accuracy rates for surgical oncologists versus other clinicians. McNemar’s test, *p < 0.05,* ns* non-significant

When comparing overall results between surgical oncologists and other clinicians, surgical oncologists achieve better results (Fig. 4). Sensitivity rates were higher in the surgical oncologists group: 61.1% (95% CI 55.6–66.2) versus 51.4% (95% CI 46.3–56.4) (*p* < 0.05). Specificity rates were higher as well: 39.0% (95% CI 31.5–47.1) versus 31.4% (95% CI 25.1–38.4) (*p* < 0.05). Accuracy rates were higher but the difference was not statistically significant: 54.2% (95% CI 49.6–58.6) versus 44.7% (95% CI 40.6–48.8) (*p* = 0.47).

## Discussion

In this study, clinicians’ performance in detecting mesocolic SLNs with ICG in intraoperative videos was assessed by comparing standard and contrast-enhanced views in a fully paired design. The results demonstrated a significant improvement in the sensitivity and accuracy of SLN detection overall, as well as across all subgroups, when using the contrast-enhancing software. Surgical oncologists achieved the best outcomes in both views. The contrast enhancement likely aided in identifying NIRF hotspots, thereby enhancing the detection of SLNs. This improved detection corresponded with increased confidence in the annotations as compared to the standard videos. However, the contrast enhancement may have contributed to a decrease (though not statistically significant) in specificity, as more areas were mistakenly identified as SLNs. Notably, time-to-detection was not significantly reduced with the contrast-enhanced view. However, given the short duration of video clips, the potential for reducing detection time may have been limited.

While the aim of using fluorescence during CC surgery is to enable local resections and minimize tissue removal, identifying and removing all SLNs is a priority. Thus, sensitivity is of greater clinical relevance, as it reflects the ability to correctly identify SLNs, with higher sensitivity corresponding to a lower rate of false negatives. Overlooking an SLN could result in incorrect staging, significantly impacting patient treatment and clinical outcomes. In contrast, lower specificity in this context means that the surgeon may inspect and dissect more tissue areas during surgery that ultimately turn out not to be SLNs upon closer inspection. However, this would not result in substantial tissue removal, and the effect on the patient would be minimal.

Another finding was the presence of observer-related differences in diagnostic performance, with surgical oncologists demonstrating higher sensitivity and specificity compared to other clinicians. This suggests that SLN detection is influenced by experience and may be acquired through learning, as previously reported in studies using various dyes [26–28]. Interestingly, the use of the contrast-enhanced view significantly improved the sensitivity in all subgroups, including the surgeon and resident, and even among surgical oncologists despite their extensive prior experience with SLN detection.

Furthermore, a very important finding was an overall low sensitivity, specificity, and accuracy in detecting SLNs. In this test setting, the sensitivity was below 50% with standard video clips, indicating that over half of the SLNs were not detected. Although this percentage increased to 62% with the contrast-enhanced view, it still implies that nearly 40% of patients could receive incorrect treatment due to undetected SLNs. Several factors may have contributed to these low rates. First and foremost, challenging video clips were deliberately selected in order to investigate potential differences between the standard and contrast‑enhanced views, thus resulting in lower scores. Second, as a result of the study design using prerecorded video clips, clinicians were unable to control the surgical camera themselves and consequently they could not further inspect and zoom in on regions of interest if needed. Third, the NIRF camera view made it challenging to discriminate between the colon and mesentery. In a real surgical setting, surgeons can switch between white light and NIRF modes as needed, enhancing their ability to distinguish tissue types. Fourth, the videos were presented to the clinicians without additional context, such as the location of the primary tumor. Fifth, when the clinicians had to manually stop the video to annotate SLNs, time pressure might have led to decisions different from those made in actual clinical scenarios. We expect clinicians to perform better when they are conducting the surgery themselves, have control over the camera and light settings, are aware of the tumor location and the spread of ICG into the mesentery, and are not constrained by time limitations. Importantly, in the earlier pilot study where the video clips were recorded, one or more SLNs were identified and marked without contrast-enhancing software in all patients during real-time surgery. Among these patients, two had lymph node metastases, with no false-negative SLNs reported [24]. This raises the question of whether contrast-enhancing software is necessary. While these results are encouraging, the number of patients with lymph node metastases was low, and it remains uncertain whether SLNs might have been overlooked in a series with a higher number of events. Additionally, some procedures were challenging because of significant NIRF background noise, emphasizing the value of contrast-enhancing software in improving sensitivity. PerfusionWorks can be used as an intraoperative adjunct during surgery as it does not disrupt normal surgical workflow, and the contrast-enhanced mode would therefore be particularly useful in challenging cases. Using contrast-enhancing software in larger patient series could help minimize the risk of overlooking an SLN and reduce false-negative results, which is crucial for ensuring optimal patient outcomes.

An additional limitation of this study was the exclusive use of videos recorded with the da Vinci robot, without considering other NIRF laparoscopic camera systems with different hardware and software specifications. The PerfusionWorks software does function with alternative camera systems. However, the contrast-enhancing software could potentially have a different effect on alternative camera systems and more systems could be studied to verify its effect. Moreover, only videos of SLN mapping procedures using ICG were analyzed. Antibody-based NIRF tracers such as bevacizumab-800CW or SGM-101, which are used for detection of malignant (colorectal) lesions, may inherently provide greater SLN contrast through more specific tumor binding than ICG [29–33]. However, contrast-enhancing software would still be beneficial, as background fluorescence is not expected to be fully eliminated. Future integration of artificial intelligence into adjunct (contrast-enhancing) software could assist (junior) surgeons in correctly locating SLNs, and help them do it faster. Researchers already demonstrated exactly this, even without NIRF, using only 22 surgical videos. Ideally, more data would further improve performance [34].

To the best of our knowledge, this is the first study to investigate the efficacy and application of contrast-enhancing software specifically in fluorescence-guided surgery. However, the most important limitation of this study is that it was conducted in a test setting using previously recorded videos rather than during actual surgery. The main advantage of the present study is its controlled, fully paired design with randomized video clips that allowed the most unbiased head-to-head comparison of standard and contrast-enhanced views. This is essential because migration of ICG to higher echelon lymph nodes occurs within minutes and could otherwise substantially influence results. Future prospective studies should focus on the real-time application of contrast-enhancing software and its impact on SLN detection and biopsy outcomes during CC surgery with consideration that the software would be an adjunct to normal workflow. We envision a prospective paired, within-patient design in which SLNs would first be marked with the standard NIRF view, after which the contrast-enhancing software is activated before resection to identify any additional fluorescent hotspots.

## Conclusion

This study provides evidence that contrast-enhancing software can improve mesocolic SLN detection with ICG among both experienced and less experienced clinicians in a test setting. Further research is warranted to evaluate the performance of NIRF contrast enhancement in real-time intraoperative settings.

## Data Availability

The data generated and analyzed during the study are not publicly available, but are available from the corresponding author on reasonable request.

## Abbreviations

CC: Colon cancer
ICG: Indocyanine green
NIRF: Near-infrared fluorescence
SLN: Sentinel lymph node
